# UNDERSTANDING MOTIVATIONS FOR PARTICIPATING IN EVIDENCE-BASED FALL PREVENTION PROGRAMS ON ZOOM

**DOI:** 10.1093/geroni/igac059.2695

**Published:** 2022-12-20

**Authors:** Andy Rapoport, Margaret Danilovich

**Affiliations:** CJE SeniorLife, Chicago, Illinois, United States; CJE SeniorLife, Chicago, Illinois, United States

## Abstract

The pivot to remote delivery of NCOA evidence-based programs has increased participant reach due to eliminating geographic or transportation barriers. Despite the convenience of participation in one’s own home, challenges for helping patients adhere to behavior change remain. This paper will present data from one community-based organization’s NCOA evidence-based falls prevention program implementation of Bingocize, Tai Chi, Otago, and SAIL on Zoom. Across all programs, only 36% of people who registered for workshops attended the first session. Participants, on average, only attended 27% of the sessions in a given workshop. To explore the barriers and facilitators to attendance in order to encourage patients to change health behavior, we used a mixed-methods approach to evaluate reasons for suboptimal adherence among n=735 participants across the 4 programs. We performed sub-group analysis to examine barriers and facilitators by the self-reported attendance rate. We conducted semi-structured interviews with n=14 participants, focusing on those who had less than 50% attendance for the workshop they registered for, and also received n=234 survey responses. For participants who attended >50% of workshop sessions, class enjoyment was the most cited facilitator of attendance. However, for those who attended < 50% of workshop sessions, email reminders were the most cited attendance facilitator. Results from this project provide critical information about specific barriers that different sub-groups of older adults face regarding participation in NCOA evidence-based programs to develop strategies to help motivate patients for health behavior change.

